# Observation study of using a small dose of rituximab treatment for thyroid-associated ophthalmopathy in seven Chinese patients: One pilot study

**DOI:** 10.3389/fendo.2022.1079852

**Published:** 2023-01-18

**Authors:** Yueyue Wang, Hao Hu, Lu Chen, Haitao Zhang, Tao Yang, Xiaoquan Xu, Huanhuan Chen

**Affiliations:** ^1^ Department of Endocrinology, The First Affiliated Hospital of Nanjing Medical University, Nanjing, China; ^2^ Department of Radiology, The First Affiliated Hospital of Nanjing Medical University, Nanjing, China

**Keywords:** thyroid-associated ophthalmopathy, treatment, rituximab, small dose, magnetic resonance imaging

## Abstract

**Objective:**

To report the efficacy, long-term safety, and tolerability of using a small dose (125 mg/m2 weekly for 4 weeks) of rituximab to treat Chinese patients with thyroid-associated ophthalmopathy (TAO).

**Methods:**

Seven patients with active moderate-to-severe TAO were prospectively recruited in this study. A small dose of rituximab (125mg/m2 body surface area) was given weekly with a duration of four weeks. Thyroid function, thyrotropin receptor antibody (TRAb), B cell and T cell subsets, ophthalmological examination, magnetic resonance imaging derived parameters, and adverse reactions were recorded at each visit.

**Results:**

Seven patients were followed for an average of 224 weeks. B-cell depletion was observed in all patients following rituximab infusion. The clinical activity score (CAS) decreased from 4.86 ± 0.69 to 3.00 ± 0.82 at 5 weeks after treatment (*P* = 0.033) and remained significantly lower than baseline values at the end of follow-up (*P* = 0.001). Compared to baseline values, significant decreases in exophthalmos of the right eye, the thickness of extraocular muscles with maximum signal intensity, and the highest signal intensity ratio (SIR) of extraocular muscle to ipsilateral temporal muscle values were observed at the last follow-up (all *P* < 0.05). Disease progressions or recurrences were not observed during follow-up. Only mild fatigue was observed after the first infusion as a side effect (n = 1).

**Conclusion:**

Small dose of rituximab may be a promising option with adequate safety, tolerability, and long-term efficacy for patients with active moderate-to-severe TAO.

## Introduction

Thyroid-associated ophthalmopathy (TAO) is the most common and serious extra-thyroid manifestation of Graves’ disease. Signs and symptoms of active TAO include eyelid contracture, exophthalmos, diplopia, corneal ulcerations, and even loss of vision (1). Intravenous glucocorticoids (GC) therapy is suggested as a first-line treatment for active and moderate-to-severe TAO. However, a proportion of patients cannot achieve remission and are defined as refractory TAO (2). Besides that, high-dose GC therapy is not always suitable for all TAO patients due to the contraindications and complications (e.g., weight gain, diabetes, high blood pressure, peptic ulcer, femoral head necrosis) (3). Thus, besides the conventional intravenous GC treatment, finding an effective alternative treatment strategy for patients with TAO was needed in clinical practice.

Although detailed pathogenesis has not been fully elucidated, the immunologic cross-activity between thyroid and orbital tissue antigens is deemed to play an important role in the occurrence and progress of TAO (4). Thyroid-stimulating hormone receptor (TSHR) is the most common pathogenic antigen in TAO (5). B cells in affected tissues can recognize TSHR and produce insulin-like growth factor-1 receptor (IGF-1R). The combination of TSHR and IGF-1R on the orbit releases cytokines, recruiting more immune cells into the orbit, causing hyaluronic acid accumulation, and expansion of orbital adipose tissue which contributes to the development of TAO (6). Teprotumumab is a complete human IgG1kappa monoclonal antibody that targets insulin-like growth factor I receptor (IGF-IR). It can reduce hyaluronan production and cytokine stimulation, thus can effectively control inflammation, and improve the exophthalmos and diplopia of patients (7). However, the expensive medical cost and uncertainty of long-term efficacy preclude its wide application. Therefore, B cells deserve consideration as a promising new therapeutic target in TAO.

Rituximab (RTX) is a chimeric human and mouse monoclonal antibody, which is expressed on pre-B cells and mature B cells. It has been proven to be useful in the treatment of autoimmune diseases such as rheumatoid arthritis, membranous nephropathy, and antineutrophil cytoplasmic antibody (ANCA)-associated vasculitis (8–10). Recently, increasing results have been reported on the RTX treatment of TAO (11–15), however, a high dose of RTX (such as 500 mg or 1000 mg twice, 2 weeks apart, 375 mg/m^2^ weekly for 4 weeks) is usually used, and therefore side effects (e.g., infusion reactions, arthralgias, optic neuropathy, abdominal pain) have been reported in about one-third of patients (16–18). Given this, some studies have tried to use low doses of RTX in eliminating B cells and reducing inflammation (14, 19). Du et al. treated 15 patients with refractory TAO using low-dose RTX (cumulative dose, 100-400mg), and clinical improvement was achieved in 87% of the patients within 2 months (14). Insull et al. found that treatment with 100 mg RTX in combination with glucocorticoids (mean dose 2.3g) or other immunosuppressive agents (methotrexate or ciclosporin) was effective in reducing clinical activity in 12 TAO patients (19). However, the follow-up period was limited in above mentioned studies, therefore the long-term effect of a small dose RTX treatment in TAO patients was still unclear.

Therefore, the purpose of this study was to investigate the long-term efficacy, safety, and tolerability of using a small dose (125 mg/m^2^ weekly for 4 weeks) of rituximab to treat Chinese patients with TAO.

## Materials and methods

### Patients

The study was approved by the ethics committees of our hospital (No. 2011-SR-032). Written informed consent was all obtained before patients recruit. Inclusion criteria were as follows: (1) age ranged from 18 to 75 years; (2) active moderate-to-severe disease defined according to the clinical activity score (CAS) (CAS ≥ 3/7) and European Group on Graves′ Orbitopathy (EUGOGO) severity assessment (20, 21); (3) normal or near-normal thyroid function (no more than twice the upper limit of normal); (4) with evidence of disease progression during the previous 2 months or no improvement in the past 6 months; (5) discontinue previous steroid treatment for at least 3 months. (6) no contraindications to MRI scanning. Exclusion criteria were as follows: (1) vision-threatening TAO; (2) medically unfit to receive RTX (history of pulmonary tuberculosis, hepatitis B carrier, hepatitis C positive, human immunodeficiency virus (HIV), absolute neutrophil count < 1.5×10^9^/L); (3) pregnant or breastfeeding.

### Study design

All individuals were treated with RTX weekly infusions of 125 mg/m^2^ of body surface area for four weeks, and dexamethasone (5 mg) was given before dosing to prevent possible allergic reactions. During the infusion, heart rate and blood pressure were monitored. Patients were evaluated before and after treatment at designated follow-up visits (5, 8, 16, 28, 52, and 224 weeks) by laboratory tests, clinical manifestation, and ophthalmic examination. Orbital magnetic resonance imaging (MRI) was performed before treatment, and at 52, 224 weeks after RTX injection. Adverse events that were reasonably or probably related to RTX were documented throughout the study period.

### Clinical assessments

Baseline assessment included medical history, concomitant medications, physical examination (by an endocrinologist with 10 years of experience), ophthalmic evaluation including proptosis, visual acuity, and intraocular pressure (by an ophthalmologist with 5 years of experience), orbital MRI, and laboratory data. The laboratory data included the thyroid-stimulating hormone (TSH), free triiodothyronine (FT_3_) free thyroxine (FT_4_), thyrotropin receptor antibody (TRAb), lymphocyte subsets (CD3^+^, CD3^+^CD4^+^, CD3^+^CD8^+^, CD19^+^, and CD20^+^), and serum immunoglobulin (IgG, IgA, IgM).

### Imaging techniques and analysis

MRI scans were performed on a 3.0-T MRI system (Magnetom Skyra; Siemens Healthcare, Erlangen, Germany) with a 12-channel head coil. Patients were instructed to take a comfortable supine position with eyes closed to reduce motion-related errors. Imaging protocols included axial T1- weighted image (repetition time/echo time, 635/6.7 ms), and axial, coronal, and sagittal T2-weighted image with fat suppression (FS) (repetition time/echo time, 4000/75–117 ms). Image analysis was performed by two dedicated radiologists (with 10 and 3 years of experience on head and neck radiology, respectively). Specific MRI-derived parameters and their measurement methods were showed as follows: (1) Exophthalmos: vertical distances from the apex of cornea to the interzygomatic line on axial T2-weighted image with FS. (2) The thickness of orbital fat (OF): the maximum distance between the medial wall of the eyeball and the medial wall of the orbit on axial T1-weighted image was measured. (3) The highest signal intensity ratio (SIR) of extraocular muscle to ipsilateral temporal muscle: on the coronal image of T2WI temporal FS, the signal intensity of extraocular muscle and ipsilateral temporal muscle with the highest T2 signal was measured by region of interest (ROI) method, and the signal intensity ratio of extraocular muscle and temporal muscle was taken as SIR. (4) The volume of extraocular muscle (EOM): the cross-sectional area of the extraocular muscle with the highest T2 signal was measured using the calculation method reported in a previous study (22). The superior rectus must be assessed along with the levator palpebrae because of the difficulty in separating them on the magnetic resonance images. The volume of EOM was obtained by the sum of the cross-sectional areas with a slice thickness of 3.5mm. The average of the measurements of two radiologists was adopted for future statistical analysis.

### Statistical analyses

Statistical analyses were performed using the SPSS software (version 23.0, SPSS, Inc., Chicago, IL) and GraphPad Prism (version 8.0.0, GraphPad Software, Inc. San Diego, CA). Continuous data were presented as the mean ± standard deviation. The repeated measures analysis of variance (ANOVA) was applied for the comparison of data at different time points. Statistical significance was defined as a two-sided *P* value less than 0.05.

## Results

### Patients

Seven patients (two males, and five females; age range of 43-62 years) were finally enrolled in the study. Among all patients, two male patients had a long history of smoking before treatment, and one female patient occasionally had a history of passive smoking. One patient had quit smoking before the treatment of rituximab, and the other patient could not quit smoking. None of the other patients had active or passive smoking during the follow-up period. Baseline patient characteristics are displayed in **Table 1**. At the time of RTX treatment, three patients were subclinical hyperthyroid, and two of them received methimazole treatment. Three were euthyroid, and one of whom was treated with levothyroxine. One patient developed hypothyroidism after radioactive iodine treatment and was then supplied with levothyroxine. During the follow-up period, five patients had normal thyroid function (71.4%), one patient had hypothyroid (14.3%), and one patient had hyperthyroidism followed by transient hypothyroidism (14.3%). After adjusting the drug dosage, the thyroid function of the patient returned to normal. Two patients did not receive intravenous glucocorticoids before RTX treatment: patient 2 because of uncontrolled diabetes, and patient 5 was reluctant to receive high-dose GC therapy because of concerns about side effects. Four patients were treated with glucocorticoids and discontinued three months before the treatment of RTX. The mean follow-up period was 224 weeks (range: 44-78 months).

**Table 1:** Demographics and clinical characteristics of seven patients treated with rituximab.

Patient number	1	2	3	4	5	6	7
**Age (years)**	46	55	62	59	44	43	49
**Sex (F/M)**	F	F	M	M	F	F	F
**Smoking history**	No	No	Yes	Yes	No	No	No
**Thyroid status**	Euthyroid	Euthyroid	Subclinical hyperthyroidism	Hypothyroidism	Subclinical hyperthyroidism	Subclinical hyperthyroidism	Euthyroid
**Therapy for thyroid dysfunction**	–	L-T4 after radioiodine	MMI	L-T4 after radioiodine	–	MMI	–
**Previous treatment**	Somatostatin	None	GC	GC	None	GC	GC
**Clinical response to GS**	Progression	–	Progression	Progression	–	Progression	Progression
**Duration of TAO (Months)**	7	1	1	11	1	3	9
**CAS**	5	4	5	5	4	6	5
**Involved of EOM**	All	IR	All	All	All	All	All

F, female; M, male; GC, glucocorticoid; MMI, methimazole; L-T4, L-thyroxine; CAS, clinical activity scores; EOM, extraocular muscle; IR, inferior rectus; TAO, thyroid-associated ophthalmopathy.

### Effects of RTX treatment on lymphocyte subgroup and serum immunoglobulin

**Figure 1:**
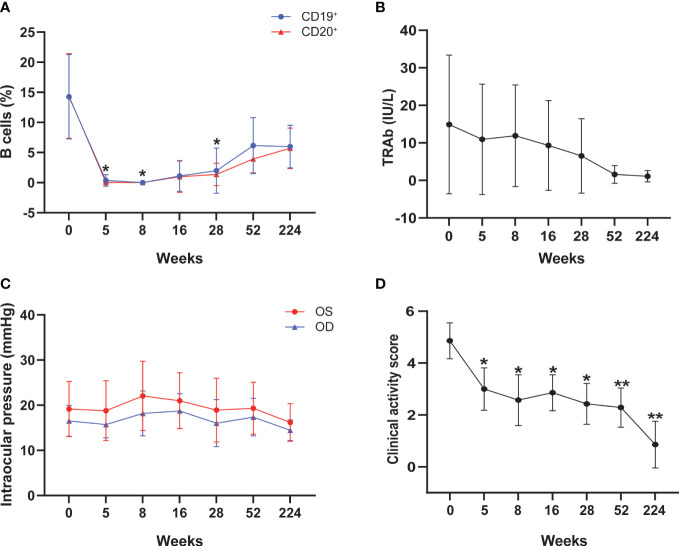
Changes of peripheral B cells **(A)**, TRAb levels **(B)**, intraocular pressure **(C)**, and clinical activity score **(D)** during the treatment of rituximab. TRAb, thyrotropin receptor antibody; The asterisk indicated significant differences (*P < 0.05, **P < 0.01).

All patients showed significant reductions in CD19^+^ and CD20^+^ cells at weeks 5 and 8 compared to baseline after RTX treatment (all *P* < 0.05), and began to increase at week 16. Until the study ended, peripheral B cells had not returned to baseline (**Table 2** and **Figure 1A**). The percentages of T lymphocyte subsets, C3 complement, C4 complement and the number of serum immunoglobulin remained stable throughout the study (**Table 2**).

**Table 2:** Changes of B and T cell subsets and immunoglobulins in TAO patients after rituximab treatment during the follow-up period.

Parameters	Time points						
	0 (baseline)	5 weeks	8 weeks	16 weeks	28 weeks	52 weeks	224 weeks
CD19	14.26 ± 7.00	0.37 ± 0.96*	0.02 ± 0.04*	1.11 ± 2.56	1.99 ± 3.74*	6.15 ± 4.63	5.99 ± 3.54
CD20	14.39 ± 7.04	0.04 ± 0.09*	0.00 ± 0.00*	0.99 ± 2.61	1.36 ± 1.86*	3.93 ± 2.32	5.70 ± 3.36
CD4 (%)	34.54 ± 10.05	35.61 ± 8.28	33.64 ± 11.62	38.80 ± 9.63	37.31 ± 12.66	36.43 ± 6.10	33.26 ± 6.97
CD8 (%)	25.37 ± 7.44	28.59 ± 6.89	28.22 ± 9.59	30.68 ± 4.47	27.33 ± 12.07	25.92 ± 5.86	21.85 ± 5.31
CD3 (%)	62.98 ± 14.92	67.99 ± 14.18	66.76 ± 19.76	72.59 ± 10.71	66.91 ± 24.85	65.93 ± 9.53	58.96 ± 10.21
IgG (g/L)	10.86 ± 2.00	12.32 ± 2.14	12.80 ± 2.85	12.21 ± 1.98	12.17 ± 1.72	12.61 ± 1.57	14.89 ± 1.60
IgA (g/L)	1.91 ± 0.69	2.10 ± 0.75	2.12 ± 0.69	2.09 ± 0.84	2.11 ± 0.74	2.14 ± 0.87	2.33 ± 0.70
IgM (g/L)	1.12 ± 0.38	1.20 ± 0.47	1.17 ± 0.43	1.16 ± 0.43	1.11 ± 0.47	0.91 ± 0.34	1.02 ± 0.38
C3 (g/L)	0.98 ± 0.28	1.07 ± 0.25	1.00 ± 0.27	1.00 ± 0.27	1.03 ± 0.22	1.02 ± 0.26	1.08 ± 0.27
C4 (g/L)	0.26 ± 0.09	0.25 ± 0.09	0.29 ± 0.09	0.27 ± 0.10	0.26 ± 0.08	0.25 ± 0.06	0.26 ± 0.07

The numeric data are reported as the mean ± standard deviation.

IgA, Immunoglobulin A; IgG, Immunoglobulin G; IgM, Immunoglobulin M; C3, C3 complement; C4, C4 complement.

*P < 0.05 versus baseline values.

### Effect of RTX treatment on thyroid function and ophthalmic indexes

No significant differences were observed in FT_3_, FT_4_, and TSH levels from baseline to each follow-up time point (**Table 3**). TRAb showed an insignificant downward trend throughout the follow-up period (**Table 3** and **Figure 1B**). Meanwhile, an increase in intraocular pressure was observed at week 8 after RTX treatment, followed by a decline, but there was no statistical difference (**Table 3** and **Figure 1C**). At the end of follow-up, the visual acuity of the patient’s right eye was significantly improved compared with that before treatment (*P* = 0.037) (**Table 3**).

**Table 3:** Changes of clinical and laboratory parameters in TAO patients after rituximab treatment during the follow-up period.

Parameters	Time points						
	0 (baseline)	5 weeks	8 weeks	16 weeks	28 weeks	52 weeks	224 weeks
FT3 (pmol/L)	5.54 ± 3.17	6.62 ± 3.63	5.68 ± 3.14	4.63 ± 0.70	3.66 ± 1.30	4.69 ± 0.41	5.24 ± 1.00
FT4 (pmol/L)	17.08 ± 4.83	18.36 ± 3.84	20.15 ± 8.10	18.69 ± 6.62	14.75 ± 7.20	16.37 ± 2.89	19.47 ± 5.89
TSH (mIU/L)	8.39 ± 21.33	4.08 ± 9.07	2.75 ± 5.80	1.71 ± 2.91	15.62 ± 37.22	3.45 ± 4.37	6.38 ± 14.26
TRAb (IU/L)	14.92 ± 18.48	10.94 ± 14.72	11.89 ± 13.55	9.32 ± 11.96	6.54 ± 9.92	1.58 ± 2.33	1.11 ± 1.51
CAS	4.86 ± 0.69	3.00 ± 0.82*	2.57 ± 0.98*	2.86 ± 0.69*	2.43 ± 0.79*	2.29 ± 0.76**	0.86 ± 0.90**
IOP (OD)	16.51 ± 3.40	15.73 ± 2.94	18.19 ± 4.98	18.70 ± 3.84	16.03 ± 5.21	17.36 ± 4.14	14.46 ± 2.28
IOP (OS)	19.16 ± 6.09	18.77 ± 6.64	22.06 ± 7.68	20.99 ± 6.18	18.93 ± 7.07	19.33 ± 5.75	16.19 ± 4.15
Visual acuity (OD)	0.71 ± 0.31	0.74 ± 0.27	0.77 ± 0.24	0.77 ± 0.24	0.77 ± 0.24	0.77 ± 0.24	0.89 ± 0.23
Visual acuity (OS)	0.71 ± 0.27	0.71 ± 0.27	0.79 ± 0.22	0.79 ± 0.22	0.79 ± 0.22	0.80 ± 0.20	0.93 ± 0.13*

The numeric data are reported as the mean ± standard deviation.

FT3, free triiodothyronine; FT4, free thyroxine; TSH, thyroid-stimulating hormone; TRAb, thyrotropin receptor antibody; IOP, intraocular pressure; OD, right eye; OS, left eye; CAS, clinical activity scores;

*P < 0.05, **P < 0.01 versus baseline values.

### Effect of RTX treatment on disease activity

The mean CAS was 4.86 ± 0.69 at baseline, decreased to 3.00 ± 0.82 at 5 weeks (*P* = 0.033), and remained at low levels throughout the follow-up period. At the end of the follow-up, the mean CAS value (0.86 ± 0.90) was still significantly lower than the initial value (*P* = 0.001) (**Table 3** and **Figure 1D**). Among those 7 patients, 5/7 (71.4%) had disease inactivation (CAS<3) at week 28, and 6/7 (85.7%) were inactive at week 52. There were no cases of relapse at 224 weeks of follow-up.

### Effect of RTX treatment on imaging parameters

**Figure 2:**
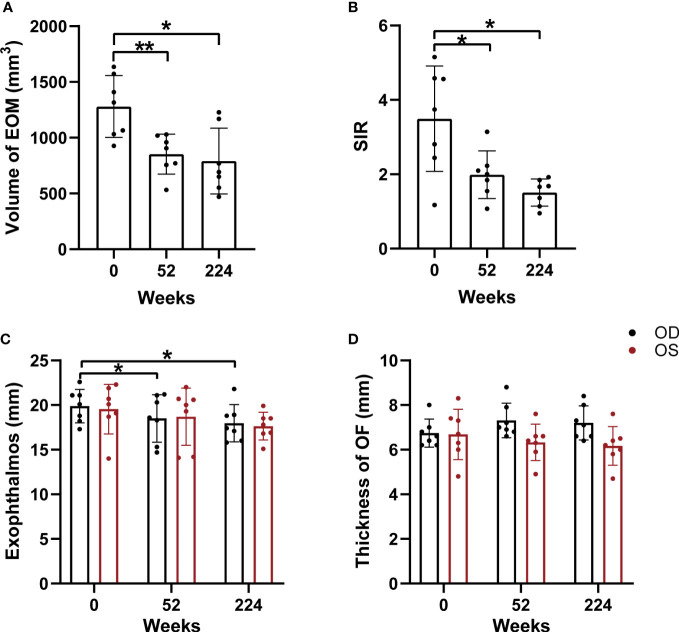
Comparisons of orbital MRI-based parameters during follow-up, including the volume of extraocular muscles with maximum signal intensity **(A)**, the highest SIR of extraocular muscle to ipsilateral temporal muscle **(B)**, the exophthalmos **(C)** and the thickness of orbital fat **(D)**. EOM, extraocular muscle; OF, orbital fat; OD, right eye; OS, left eye; SIR, the highest signal intensity ratio of extraocular muscle to ipsilateral temporal muscle. Asterisk indicated significant differences (*P < 0.05, **P < 0.01).

**Figure 3:**
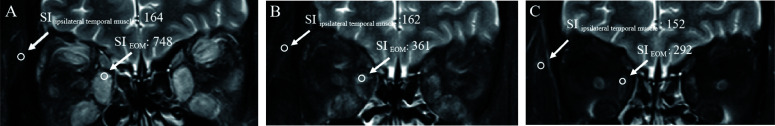
Orbital images of a patient with TAO at baseline **(A)**, 52 weeks **(B)**, and 224 weeks **(C)** after rituximab treatment. EOM, extraocular muscle; SI: signal intensity.

The volume of extraocular muscles with maximum signal intensity before therapy was 1279.65 ± 277.07 mm^3^, which decreased to 853.15 ± 178.54 and 790.46 ± 295.30 mm^3^at 52 weeks after treatment and the last follow-up (*P* = 0.003 and 0.015, respectively) (**Table 4** and **Figure 2A**). The highest SIR of extraocular muscle to ipsilateral temporal muscle values significantly decreased at 52 weeks (1.989 ± 0.639) and the last follow-up (1.508 ± 0.364), compared to those observed at baseline (3.495 ± 1.420) (*P* = 0.030 and 0.029, respectively) (**Table 4** and **Figure 2B**). In addition, the exophthalmos of the right eye also significantly decreased at 52 weeks and the last follow-up (all *P* = 0.013) (**Table 4** and **Figure 2C**). We did not observe significant changes in the exophthalmos of the left eye and the thickness of orbital fat after RTX treatment (all *P* > 0.05) (**Table 4** and **Figures 2C, D**). **Figure 3** shows the MRI images of SIR in a representative case at pre-treatment and follow-up examinations.

**Table 4:** Changes of imaging parameters in TAO patients after rituximab treatment during the follow-up period.

Parameters	Time points			*P*-value	
	0 (baseline)	52 weeks	224 weeks	*P*_0-52_	*P*_0-224_
Exophthalmos (OD)	20.51 ± 1.89	18.93 ± 2.27	17.97 ± 2.08	0.013*	0.013*
Exophthalmos (OS)	20.17 ± 2.48	18.83 ± 2.05	17.64 ± 1.54	0.345	0.093
OF (OD)	6.74 ± 0.63	7.31 ± 0.78	7.20 ± 0.76	0.133	0.087
OF (OS)	6.69 ± 1.13	6.33 ± 0.82	6.17 ± 0.87	0.305	0.226
SIR	3.50 ± 1.42	1.99 ± 0.64	1.51 ± 0.36	0.030*	0.029*
EOM (mm^3^)	1279.65 ± 277.07	853.15 ± 178.54	790.46 ± 295.30	0.003**	0.015*

The numeric data are reported as the mean ± standard deviation.

OD, right eye; OS, left eye; OF, the thickness of orbital fat; EOM, the volume of extraocular muscle with maximum signal intensity. SIR, the highest signal intensity ratio of extraocular muscle to ipsilateral temporal muscle.

*P < 0.05, **P < 0.01 versus baseline values.

### Side effect

Only one patient experienced fatigue after the first infusion, but it did not recur in subsequent infusions. There were no other side effects of the RTX infusions during a 224-week follow-up observation period.

## Discussion

In this study, we found that a low dose sustained RTX (125 mg/m^2^ weekly for 4 weeks) was feasible and effective for Chinese patients with TAO. Four of seven TAO patients were refractory to GC therapy before RTX treatment, however marked improvement was obtained within a very short period after RTX infusion. It is noteworthy that none of the patients experienced late recurrence during a long-term follow-up period of more than 4 years. To our knowledge, our study was the first one reporting the effectiveness of a small dose of RTX in achieving effective peripheral B-cell depletion and long-term remission of TAO in the Chinese population.

Most of the previous studies used RTX by the protocol of rheumatoid arthritis protocol (1 g with 2-week intervals) or lymphoma (375 mg/m^2^ weekly for 4 weeks) (12, 23–25). However, the optimal dose of RTX has not been clarified. Previously, Maloney et al. reported that the use of weekly infusions times four of 125 mg/m^2^, 250 mg/m^2^, and 375 mg/m^2^ of RTX in patients with relapsed lymphoma could cause B-cell depletion (26). In autoimmune disease, where the prognosis is less severe than in lymphoproliferative disease, we consider lower doses for TAO patients. Our results suggest that B cells were rapidly and effectively depleted by RTX injection at 125kg/m^2^ per week for 4 weeks.

An important finding from our study is that sustained long-term remissions can be achieved in patients with TAO after this small dose of RTX treatment. A previous study found that 5 patients with refractory TAO following a 4-week course of weekly rituximab (375 mg/m^2^), and the CAS decreased significantly within one month, but there was no further change in the subsequent 5 years of follow-up (17). In our study, a decrease in CAS was observed in all patients at the first week after treatment and remained stably low during the follow-up, with a further significant decrease from baseline after an average of 224 weeks of follow-up. The observed improvement in CAS seemed to correlate with the onset of B-cell depletion (23). It is worth noting that we did not observe disease relapse after B cell reconstitution. Thus, these findings offer strong clinical evidence of the usage of small dose of RTX for TAO treatment.

Though a downward trend was observed in TRAb after the whole follow-up course, it was not statistically significant. Our findings are consistent with previous studies that showed no significant effect on TRAb with either low-dose or high-dose RTX treatment (13, 27). This might be due to RTX targeting only immature B cells, leaving the mature plasma cells that produce TRAb unharmed (28). Conversely, some studies have shown a significant reduction of TRAb in RTX-treated patients (11, 29). These studies suggested that the changes in TRAb may be attributed to the direct effect on TRAb or remission of hyperthyroidism after long-term antithyroid drug therapy. None of the seven patients had a recurrence of ocular inflammation after RTX treatment, despite the fact that the drug did not affect TRAb in our study. These initial findings warrant further investigation to fully elucidate the underlying mechanism.

Additionally, this study presented that the right eye protrusion of TAO patients decreased significantly after treatment. Some studies have shown similar results (15), whereas others reported no effect of RTX on proptosis and can cause a transient increase in protrusion (11, 30). The similarities of these studies are the use of large doses of RTX (1000 mg twice), Stan and Salvi have pointed out that high-dose of RTX can cause an increase in the volume of orbital tissue and proptosis in some patients, followed by volume displacement due to massive dissolution of B cells and may increase the risk of dysthyroid optic neuropathy (DON) (18). This can also explain the transient increase in intraocular pressure observed at week 8 of our study, accompanied by the complete depletion of B cells. Although no changes in protrusions were observed under a very low dose of RTX (100mg), the occurrence of DON cannot be avoided and patients required prompt surgical orbital decompression (19, 31). By contrast, no such case was noted in our study. In addition, we found that a small dose of RTX had a long-term positive effect on visual acuity in patients with TAO, although with the limitation of small subjects. The results of the 224 weeks of data on the patients that continued per protocol suggest the RTX dose of 125 mg/m^2^ weekly for 4 weeks may prevent the progression of DON. This is a promising finding, given that the optimal dose has not as yet been fully determined, further research with a larger sample size is warranted to confirm the findings.

In this study, we applied an objective approach to dynamically evaluate the long-term efficacy of RTX treatment in TAO patients. The increased signal intensity of enlarged EOM on T2-weighted images and the volume of EOM reflected the edematous changes in the active inflammation stage (22, 32). It has been previously confirmed that SIR of extraocular muscle to temporalis muscle showed significant positive correlations with CAS values, and could be used as an indicator to evaluate the inflammation activity of TAO (33). We observed a significant decrease in SIR and the volume of EOM with maximum signal intensity at weeks 52 and 224 after RTX treatment, compared to the baseline value. In addition, the thickness of EOM with maximum signal intensity was significantly reduced from baseline to the end of follow-up. These results highlight the ability of a small dose of RTX to produce long-term sustained relief of inflammatory edema in patients with TAO.

However, our analysis may have been limited by several factors. First, small numbers of subjects, non-randomized studies, and the lack of a double-blind, placebo-controlled design. Second, the favorable long-term outcomes seen after RTX treatment cannot rule out the influence of the natural history of the disease. Further well-designed and large-scale randomized controlled studies are needed to determine the optimal dose regimen, long-term benefits, and possible side effects. Finally, only the changes in clinical serological, immunological, ophthalmological, and imaging indicators were analyzed. Further research on the assessment of patients’ quality of life (QOL), may provide clinicians with important information about the psychosocial functions of those patients, which will help to understand more comprehensively the impact of RTX on patients with TAO.

## Conclusions

In our study, we found that a small dose of RTX (125 mg/m^2^ weekly for four doses) was safe and long-term efficacious for patients with TAO. Besides the conventional intravenous GC treatment, a small dose of RTX might be an alternative treatment strategy for patients with active moderate-to-severe TAO.

## Data availability statement

The original contributions presented in the study are included in the article/supplementary material. Further inquiries can be directed to the corresponding authors.

## Ethics statement

The studies involving human participants were reviewed and approved by the First Affiliated Hospital of Nanjing Medical University (No. 2011-SR-032). The patients/participants provided their written informed consent to participate in this study.

## Author contributions

Conceptualization, YW; methodology, YW and HH; investigation, LC; resources, TY; data curation, HZ; writing-original draft preparation, YW; writing-review and editing, HC and XX; supervision, HC. All authors contributed to the article and approved the submitted version.
